# Accumulation patterns of anthocyanin and γ-oryzanol during black rice grain development

**DOI:** 10.1371/journal.pone.0302745

**Published:** 2024-05-22

**Authors:** Manisha Thapa, Lei Liu, Bronwyn J. Barkla, Tobias Kretzschmar, Suzy Y. Rogiers, Terry J. Rose

**Affiliations:** 1 Faculty of Science and Engineering, Southern Cross University, Lismore, New South Wales, Australia; 2 NSW Department of Primary Industries, Wollongbar, New South Wales, Australia; 3 Centre for Organics Research, Southern Cross University, Lismore, New South Wales, Australia; Hainan University, CHINA

## Abstract

Pigmented rice, especially black rice, is gaining popularity as it is rich in antioxidants such as anthocyanins and γ-oryzanol. At present, knowledge about temporal control of biosynthesis and accumulation of antioxidants during grain development is limited. To address this, the accumulation patterns of anthocyanins and γ-oryzanol were assessed in two distinct black rice genotypes over the course of grain development, and the expression of known regulatory genes for anthocyanin biosynthesis was examined. The results indicated that total γ-oryzanol content increased continuously throughout grain development, while total anthocyanins peaked at dough stage (15 to 21 days after flowering) followed by a decline until grain maturity in both genotypes. However, the rate of decrease in anthocyanin content differed between genotypes, and a more prominent decline in cyanidin 3-*O*-glucoside (C3G) relative to peonidin 3-*O*-glucoside (P3G) was observed for both. Anthocyanin content was closely linked with the expression of key regulatory genes in the MBW (MYB-bHLH-WD40) complex. This improved knowledge of the genotype-specific biosynthesis (anthocyanins only) and accumulation patterns of anthocyanins and γ-oryzanol can inform subsequent research efforts to increase concentrations of these key antioxidants in black rice grains.

## Introduction

Rice (*Oryza sativa* L.) is one of the world’s major food crops, contributing more than 20% of global calorie intake [1]. Domesticated white rice arose from a loss-of-function mutation of the Rc locus in wild rice, which is primarily red in colour [2]. On the other hand, black rice resulted from a gain-of-function mutation in the Kala4 locus of cultivated rice [3]. Although cultivated whole-grain rice comes in various colours (white, brown, red, black, and purple), it is mainly consumed as polished white rice by removing the fibrous bran layer from brown rice [2]. However, whole grain black/purple rice is gaining popularity amongst consumers due to its high concentration of plant secondary metabolites including anthocyanins and γ-oryzanol, both beneficial for human health [4].

At least 18 anthocyanins have been reported in pigmented rice grains, with cyanidin 3-*O*-glucoside (C3G) the most abundant, followed by peonidin 3-*O*-glucoside (P3G). Together these account for more than 80% of the total anthocyanin content in the grain [5]. Anthocyanins play a role in human health due to their ability to act as antioxidants and to slow down the digestion of starch. Because of this activity they can promote visual acuity, prevent sclerosis, reduce cancer cell proliferation and inflammation, alleviate obesity and diabetes, and control the level of lipids in the blood [6]. At least 25 different γ-oryzanols have been identified in rice, with 24-methylene cycloartanyl ferulate, cycloartenyl ferulate, campesteryl ferulate and β-sitosteryl ferulate comprising 80% of total γ-oryzanol content [7]. In human clinical trials, supplementation with γ-oryzanol was shown to increase muscle strength and lower low-density lipoprotein cholesterol in treatment groups, with both these responses attributed to the antioxidant properties of γ-oryzanol [8].

The biosynthesis and accumulation of anthocyanins and γ-oryzanol in pigmented rice genotypes during grain development is poorly understood. Rice grain development is typically classified into five stages: i) anthesis, about 0–7 days after flowering (DAF); ii) milky stage, about 8–14 DAF; iii) dough stage, about 15–21 DAF; iv) yellow-ripe stage, about 22–28 DAF; and v) fully ripe stage, about 29–35 DAF [4]. The ideal time to harvest grain is when the grain moisture is around 20–25% or when 80–85% of the grains in the panicle are straw-yellow in colour or at the fully ripe stage [4, 9].

While some studies have shown an increase in anthocyanin concentration with grain development to maturity [4, 10], other studies found an initial increase in anthocyanin concentrations followed by a decrease as the grain nears maturity [11, 12]. Conflicting results have also been reported in respect to the accumulation patterns of γ-oryzanol during grain development. While one study found no significant difference in γ-oryzanol concentration between immature and mature rice grains [13], decreased [14] or increased [15] concentrations have also been reported. While it is possible that the reported inconsistencies for both γ-oryzanol and anthocyanin concentration in rice grain may be genotype-related, other factors such as temperature, light, UVB radiation, and nutrients may also play a role.

The regulatory and structural genes involved in anthocyanin biosynthesis have been well-defined in the rice pericarp. Core structural genes involved in anthocyanin biosynthesis include chalcone synthase (CHS), chalcone isomerase (CHI), dihydroflavonol 4-reductase (DFR), anthocyanidin synthase (ANS) and UDP-glucose: flavonoid 3-O-glucosyltransferase (UFGT) [16]. These structural genes are regulated by the MBW (MYB-bHLH-WD40) complex consisting of basic helix-loop-helix (bHLH) transcription factors (TFs), WD-repeat proteins, and the MYB-type TFs [17]. Although all three components of the MBW complexes are evolutionary conserved protein in all eukaryotes [18], different MBW complex genes are responsible for anthocyanin biosynthesis in leaves, pericarp, husks, culms and awns in rice [17]. In rice, OsKala3 and OsC1 are the MYB TFs [10] whereas OsKala4 and OsRb are the bHLH TFs controlling anthocyanin biosynthetic genes in pericarp and leaf sheath, respectively [19]. The bHLH TF is needed for transcriptional complex formation at the promoter region of various anthocyanin biosynthesis structural genes in coordination with MYB TF [10]. Although WD repeat protein seems to have no direct regulatory role in anthocyanin biosynthesis, it works as a scaffold protein that helps stabilise the interaction between MYB and bHLH TF [20]. Despite various studies investigating the exact identity and role of these regulatory components for anthocyanin biosynthesis in rice pericarp, a complete picture has only recently begun to emerge [10, 17]. Kim, Yang [10] reported OsKala3, OsKala4, and OsTTG1 as the core regulatory genes encoding the MYB TF, bHLH TF, and the WD40 repeat protein, respectively, in the rice pericarp. The expression level of these regulatory genes increased during rice grain development and correlated with the anthocyanin accumulation pattern [10].

γ-Oryzanol is a mixture of compounds formed by the esterification of ferulic acid and different phytosterols [21]. However, its biosynthetic pathway is not well-defined. Esterases which might contribute to the esterification of ferulic acid or sterols have yet to be identified in rice. Although a sterol O-acyltransferase is involved in the biosynthesis of steryl ferulates in *Arabidopsis thaliana* (L.) seeds [22], a potential role for its rice orthologs in γ-oryzanol biosynthesis remains unresolved.

The present study aimed to investigate the accumulation patterns of anthocyanins and γ-oryzanol in developing grains of two pigmented rice genotypes that differ in anthocyanin and γ-oryzanol content. The expression of key regulatory genes involved in anthocyanin biosynthesis during grain development was also examined. Due to limited information on the γ-oryzanol biosynthesis pathway and regulation, examination of regulatory genes in the present study was limited to those involved in anthocyanin biosynthesis.

## Materials and methods

### Preliminary experiment

Fourteen black rice genotypes were chosen from a black rice collection obtained from the Genetic Resource Centre of the International Rice Research Institute (IRRI), the Philippines as shown in supplementary data (S1 Table). Genotypes were chosen based on early flowering, short stature and wide range of seed colour to screen for high and low grain anthocyanin and γ-oryzanol concentrations. Selected black rice genotypes were grown in a glasshouse at NSW Department of Primary Industries, Wollongbar, under controlled night/day temperatures ranging from 20 to 30°C. Seeds were germinated on a wire mesh attached to styrofoam floating above deionised water. After 4 weeks, three developmentally similar seedlings for each genotype were transplanted into 5 L pots, with three replicate pots per genotype. All individuals were grown to maturity, and panicles were harvested at the onset of the straw-yellow stage. Harvested panicles were dried in a 40°C oven until constant weight. Rice grains were de-husked using a manual de-husker and stored in an airtight container wrapped in aluminium foil at room temperature until analysis of anthocyanin and γ-oryzanol concentrations using High-Performance Liquid Chromatography with a DAD detector as described in detail below.

### Grain development experiment

#### Plant growth

Two *japonica* black rice genotypes determined to be high and low in grain anthocyanin and γ-oryzanol concentration in the preliminary experiment were selected and grown under controlled environment conditions. Seeds were germinated as described above. After a week, the deionised water was replaced by a full-strength Yoshida solution and plants were grown for 2 weeks with the nutrient solution replaced weekly.

After 3 weeks, uniformly sized seedlings were transplanted into pots containing soil collected from the upper 0–15 cm layer of the Brookside field site at Southern Cross University’s Lismore campus (28°49′3.74″ S, 153°18′21.49″ E). Soil properties are given in S2 Table. The soil air-dried before passing through a 1 cm sieve to remove larger soil clods and foreign material. Square top 4 L pots were filled with 2.5 kg of air-dried soil, leaving 10 cm of headspace, and supplemented with 1 g of urea, 0.5 g triple super phosphate, and 0.3 g of sulphate of potash, mixed evenly throughout the pot one day before rice transplanting. One seedling was transplanted into each pot.

#### Experiment design and set up

The pot trial was conducted in a polycarbonate house at Southern Cross University, Lismore, NSW, Australia. The cladding of the house blocks 100% of outdoor UV-B radiation; therefore, two Phillips UV-B broadband (TL 40W/12 RS SLV 25, 102V, 40W) lamps were placed above the plants, maintaining a distance of 80 cm above the growing tips. The plants were exposed to the UV-B from 11 am to 2 pm daily from the tillering stage until maturity. UV-B radiation (280–320 nm) was measured using a Solarmeter model 6.2 (Solar Light Company, Inc., USA), and UV-B readings ranged from 75 to 175 μW/cm^2^ at the top of the plants. Temperature was maintained between 20°C to 32°C throughout the growing period. Rice plants were grown under flooded conditions throughout the growing period by filling the pot headspace with water as needed. Photosynthetically active radiation (PAR) (400–700 nm) ranged from 800 to 1100 μmol/m^2^/s on sunny days when readings were taken at mid-day with an Apogee MQ-500 full spectrum PAR meter.

All pots were laid out in a completely randomised design for each genotype, with each pot containing only one rice plant. These pots were further randomised every second day. Genotypes were not mixed because of differences in plant height, so as to maintain same distance from the plants to the UVB lamps throughout growing period.

#### Tagging and harvesting of grain samples

When 50% of the panicle was flowering, 42 panicles from plants of the two genotypes were tagged. Six randomly selected panicles were harvested every 5 d until maturity at 35 DAF (i.e., seven sampling points) (S1 Fig). Once a panicle was harvested from a tagged plant, no further sampling was carried out on the same plant to avoid the possible impact of source-sink changes in the remaining panicles.

At each of the seven sampling time points, tagged panicles were cut, and grains were quickly separated from panicle. In a 2 mL microcentrifuge tube, 0.5 g grain was weighed and then frozen in liquid N prior to storage at -80°C for future RNA extraction. The remaining grains were weighed and stored at -20°C in 15 mL falcon tubes to analyse anthocyanin and γ-oryzanol concentrations later.

#### Sample preparation and extraction of anthocyanin and γ-oryzanol

Stored samples were freeze-dried until constant weight. Grains were de-husked manually for samples from 5 DAF, while for samples from 10 DAF onwards, a customised manual de-husker was used to loosen the husk from whole grain and further husk removal was performed manually. Samples were ground to powder using a laboratory ball mill (Mixer ill MM301, Retsch).

A subsample of ~50 mg finely ground grain was extracted in a 15 mL falcon tube with 10 mL of methanol acidified with 1N HCl (85:15, v/v) for anthocyanin extraction and separately with 1 mL absolute methanol for γ-oryzanol extraction. The mixture was sonicated for 30 min in a Soniclean Ultrasonic cleaner (100% maximum power) at room temperature. The crude extract was centrifuged at 12,000 g for 10 min at room temperature. The resultant supernatant (~800 μl) was pipetted into 2 mL Agilent HPLC vials and stored at -20°C until analysis using High-Performance Liquid Chromatography (HPLC).

#### Analysis of anthocyanin concentration

Anthocyanin analysis was performed using an Agilent 1260 Infinity II-High Performance Liquid Chromatography instrument (Agilent Technologies, Palo Alto, CA, USA) equipped with an auto sample injector (G7129C), vacuum degasser, quaternary UHPLC 1260 flexible pump (G7104C) and diode array detector (DAD, 1260). An Agilent 1.8 μm ZORBAX Extend-C18 reverse phase column (2.1 mm internal diameter x 50 mm length) was used for the analysis. The mobile phase for anthocyanin quantification was 100% MilliQ water with 0.05% Trifluoroacetic acid (TFA) (Solvent A) and 100% acetonitrile with 0.05% TFA (Solvent B). The mobile phase gradient was 0.0–10.00 min 1 to 35% B; 10.00–10.30 min 35 to 99% B; 10.30 to 11.00 min 99% B; 11.00–11.30 min 99% to 1% B; 11.30 to 15.00 1% B at the flow rate of 0.3 mL/min. The injection volume of the sample was 3 μl, and the column temperature was set at 40°C. The UV absorbance was detected at 520 nm for anthocyanin quantification. Commercial standard cyanidin 3-*O*-glucoside (as Cl salt) was obtained from Phytolab GmbH & Co.KG, BY, Germany. All the peaks for different anthocyanins were observed at 520 nm. The lower limit of quantification for anthocyanin was 0.43 μg/mL. Only those anthocyanins which are above the quantification limit were measured. The major anthocyanin cyanidin 3-*O*-glucoside (C3G) was quantified by comparing the area under the curve for a known concentration of standard. Peonidin 3-*O*-glucoside (P3G) concentration in sample was estimated using same standard as for C3G.

#### Analysis of γ-oryzanol concentration

Total γ-oryzanol was assessed with the same HPLC instrument and column as the anthocyanins. The mobile phase used for γ-oryzanol quantification consisted of 60% acetonitrile + 40% MilliQ water in 10 mM ammonium formate and 0.1% formic acid (as solvent A), and 10% acetonitrile + 90% isopropanol in 10 mM ammonium formate and 0.1% formic acid (as solvent B). The gradient was programmed as 0.0–2.5 min, 15 to 60% B, 2.5–15.00 min 60 to 80% B, 15.00 to 16.00 min 80 to 99% B, 16.00–16.50 min 99% B, 16.50–16.60 min 99 to 15% B, 16.60 to 18.00 15% B at the flow rate of 0.3 mL/min. The injection volume of the sample was 3 μl, and the column temperature was set at 50°C. The UV absorbance was detected at 325 nm for γ-oryzanols. γ-oryzanol mix standard was purchased from Selleck Chemicals, TX, USA. The sum peak area of the sample between 7 to 9 min was compared with the sum peak area of the standard at the same time to quantify γ-oryzanol. The lower limit of quantification for total γ-oryzanol was 2.2 μg/mL.

#### RNA extraction and qPCR analysis

Total RNA was extracted from grain samples harvested at different grain development stages using Fruit-mate (Takara, Otsu, Japan, #9192) and TRIZol^TM^ Reagent for RNA extraction (Invitrogen, Carlsbad, CA, USA, #15596018). The RNA was purified using a Direct-zol^TM^ RNA Miniprep kit (Zymo Research, Irvine, CA, USA, #R2052). The first strand of cDNA was synthesised from 1 μg of total RNA using a QuantiTect Reverse Transcription kit (Qiagen, Hilden, Germany, #205313). qPCRs were performed using a QuantiNova SYBR Green PCR kit (Qiagen, Germany, #208057) and a QIAquant^TM^ real-time PCR cycler (Qiagen, Germany), using the manufacturer’s instructions. The PCR cycling was carried out under the following conditions: 2 min at 95°C, and 40 cycles of 5 s at 95°C and 10 s at 60°C in a qPCR 96-well skirted microplate (Qiagen, Germany, #209002).

Primers for three main regulatory genes (OsKala3, OsKala4, and OsTTG1) controlling the expression of the anthocyanin biosynthesis genes in rice as used by Kim, Yang [10] (S3 Table). Primers were obtained from Sigma Aldrich, Missouri, United States, along with primer for Ubiquitin (OsUBI) which was used as an internal reference to normalise the expression of all the targeted regulatory genes in the samples. Three biological and three technical replicates were used for each sample treatment. The relative expression level was calculated using the 2^-ΔΔ*CT*^ method, where data is normalised to internal reference (OsUBI) and average Ct of the gene of interest at different sampling points.

### Statistical analysis

R studio was used to calculate the means of all data and to perform one-way analysis of variance (ANOVA) for each genotype separately. Tukey’s HSD test was conducted to test the significant difference between different treatment means at 95% confidence level (p< 0.05).

## Results

### Preliminary experiment

**Fig 1:**
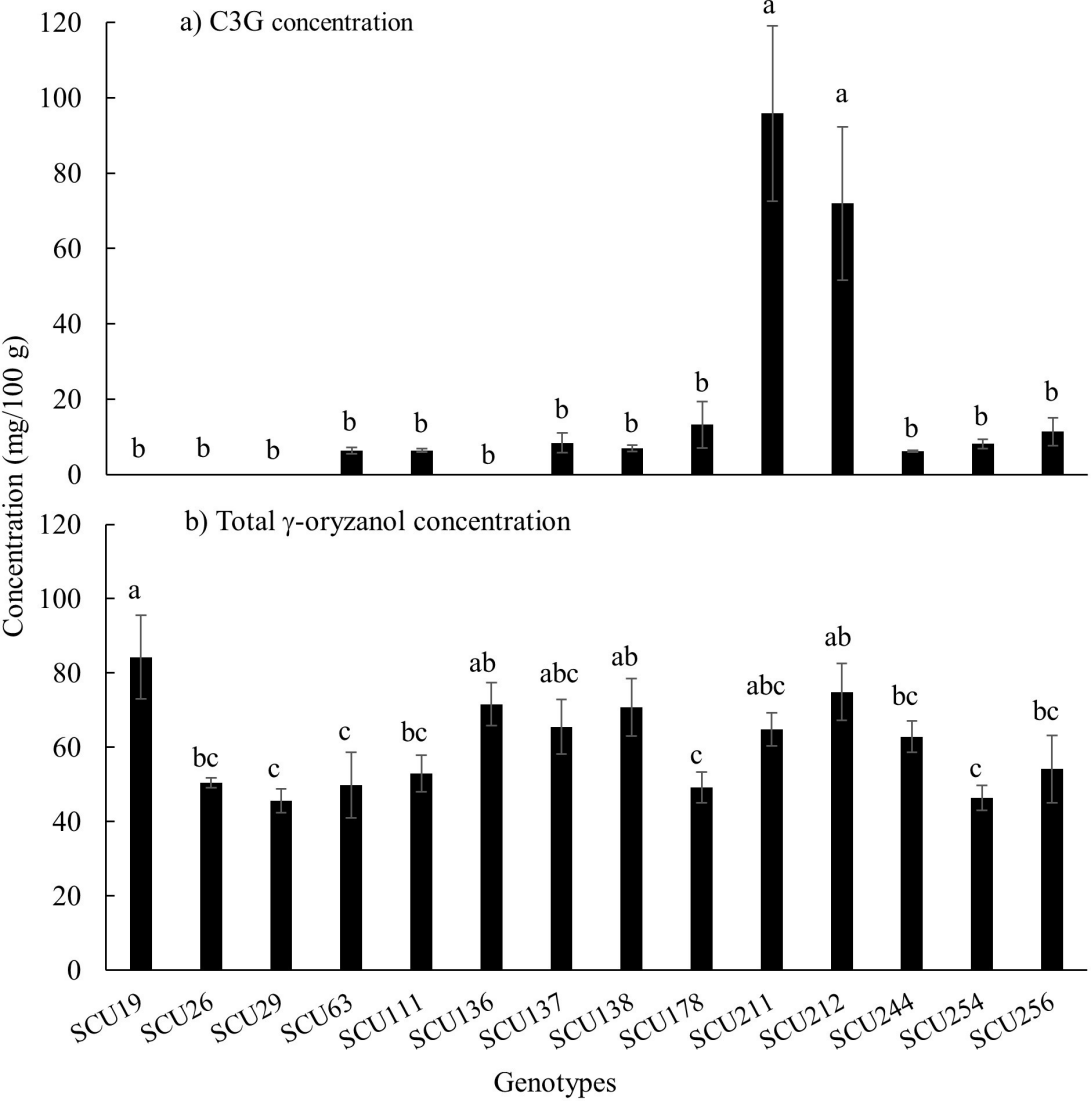
Concentration of (a) Cyanidin 3-*O*-glucoside (C3G) concentration and (b) total γ-oryzanol concentration in fourteen black rice genotypes at 50 to 60 days after flowering. Vertical error bars represent standard error (n = 3). Bars that do not share a common letter are significantly different (*p*<0.05). A mean value of zero for C3G (i.e., no bar) indicates concentrations were below the C3G detection limit of 0.43 μg/mL.

Across the fourteen rice genotypes, C3G concentration ranged from 6 to 95 mg/100 gm DW and total γ-oryzanol concentration varied between 45 to 85 mg/100 gm DW on average which was within the expected range [4, 21]. Genotypes SCU212 and SCU211 (both *japonica* subspecies) were found to be significantly higher than other genotypes in cyanidin 3-*O*-glucoside (C3G) concentration (Fig 1A). Genotype SCU212 was also significantly higher in γ-oryzanol concentration than SCU254, SCU178, SCU63 and SCU29 (Fig 1B). Genotype SCU212 was high in both C3G and γ-oryzanol concentration and therefore was selected for further experimentation. For comparison, genotype SCU254 was chosen for its low C3G and γ-oryzanol concentration (Fig 1).

### Grain-filling patterns of two distinct pigmented rice genotypes

**Fig 2:**
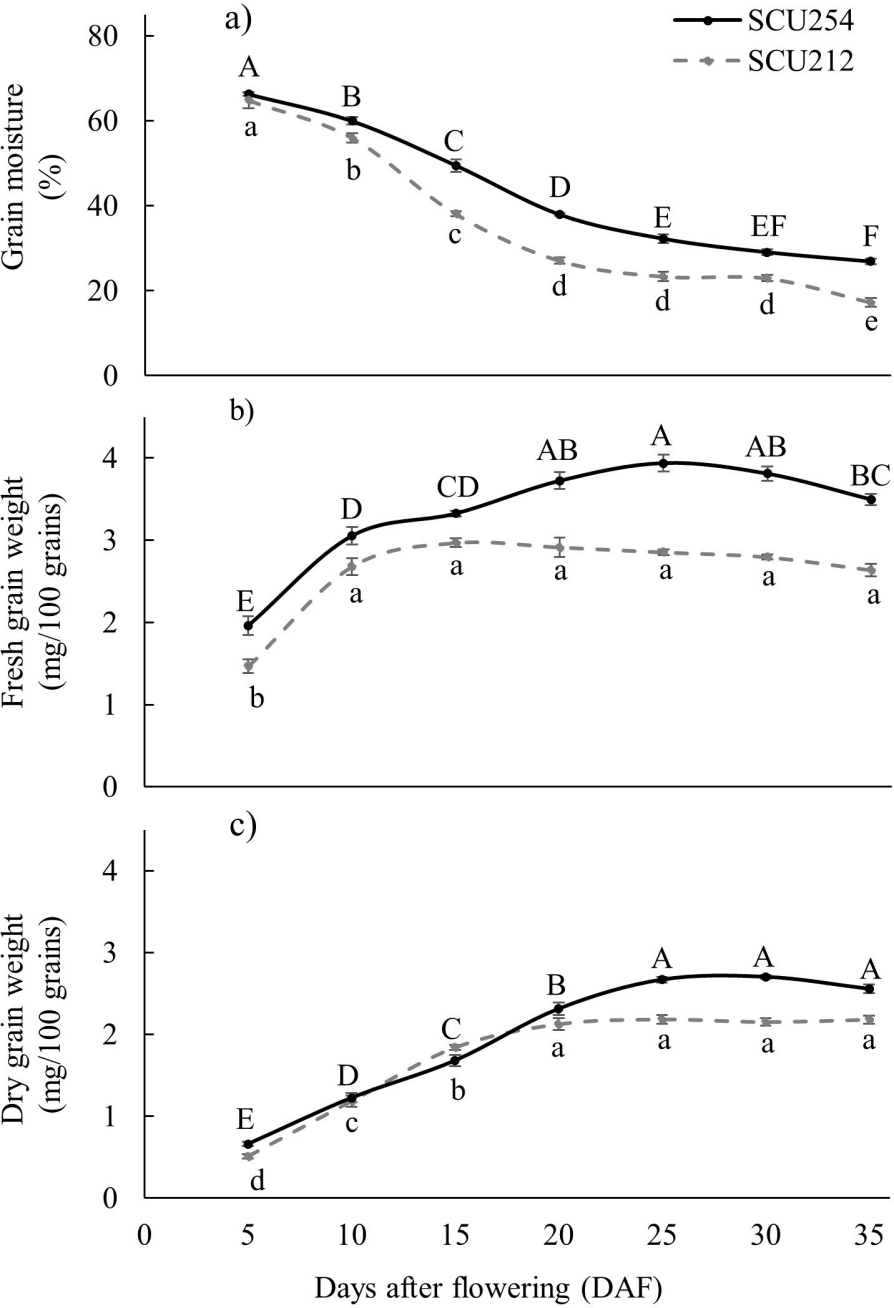
Changes in (a) Rice grain moisture content, (b) fresh grain weight, and (c) dry grain weight in genotypes SCU212 and SCU254 during grain development. Vertical error bars represent standard error (n = 6). Means for a given genotype that do not share a common letter are significantly different (*p*<0.05). Grey dotted line represents genotype SCU212 and solid black line represents genotype SCU254.

Fresh grain weight increased until 20 to 25 DAF for SCU254 (Fig 2B), whereas maximum grain fresh weight was attained as early as 10 to 15 DAF for SCU212 (Fig 2B). Maximum grain dry weight (the point after which no significant increase in weight occurred) was achieved by 25 DAF in SCU254 and 20 DAF in SCU212 (Fig 2C).

Physiological maturity of the grain is obtained at 25% to 30% moisture. This phenological stage (27% moisture) occurred at 20 DAF for SCU212 and at 35 DAF for SCU254. At maximum fresh weight, both genotypes had a similar moisture content of 38% (Fig 2A and 2B).

### Change in anthocyanins and γ-oryzanol during grain development

**Fig 3:**
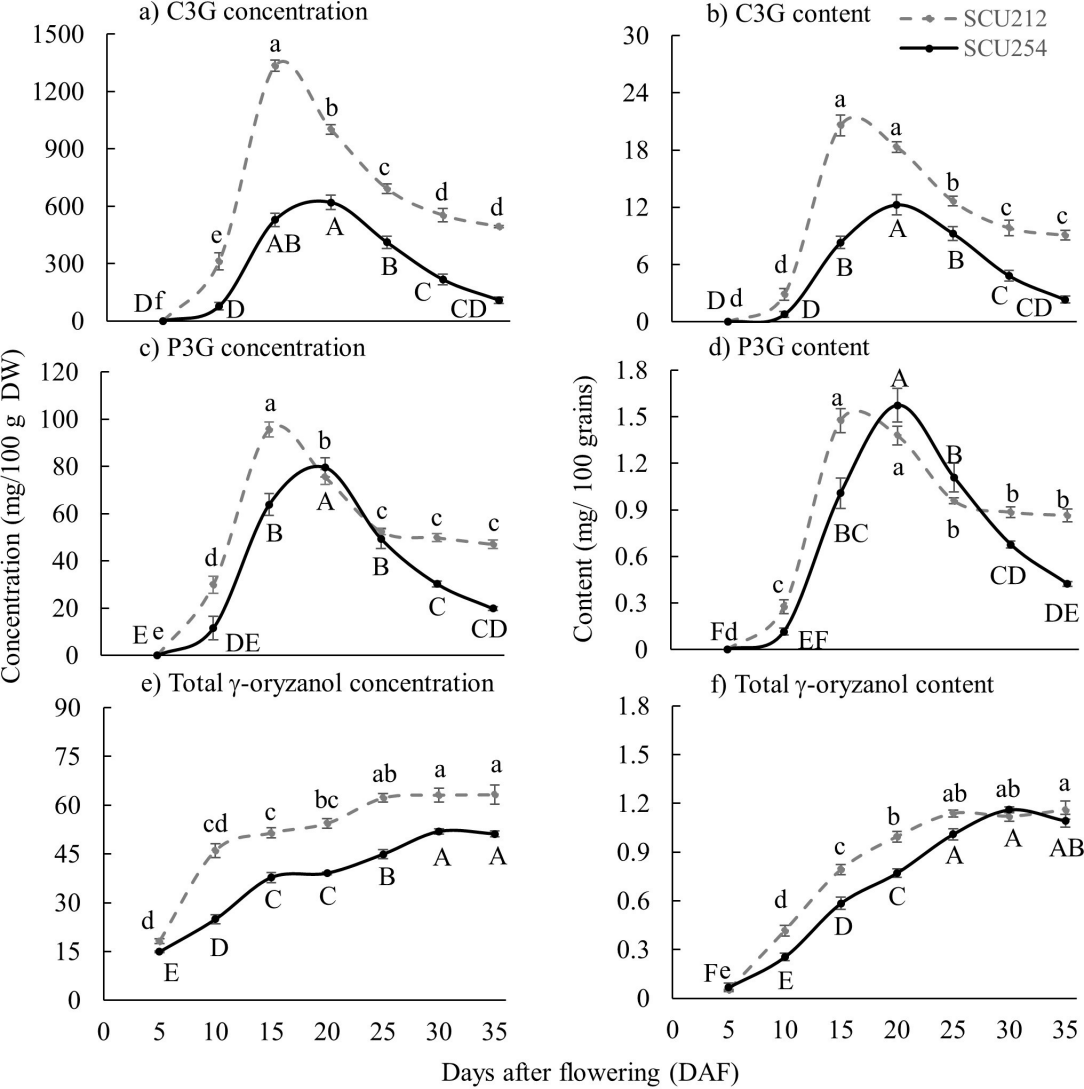
Concentration of (a) Cyanidin 3-*O*-glucoside (C3G) concentration, b) Cyanidin 3-*O*-glucoside (C3G) content, (c) Peonidin3-*O*-glucoside (P3G) concentration, (d) Peonidin 3-*O*-glucoside (P3G) content, (e) Total γ-oryzanol concentration and (f) Total γ-oryzanol content in SCU212 and SCU254 black rice genotypes in rice grain during grain development stages. Results represent mean value ± SE from six independent biological replicates. Means for a given genotype that do not share a common letter are significantly different (*p*<0.05). Grey dotted line represents genotype SCU212 and solid black line represents genotype SCU254.

Neither C3G nor P3G was detected at 5 DAF in either genotype. For both genotypes, C3G and P3G concentrations peaked at 15 to 20 DAF, then declined in the later grain development stages (Fig 3A and 3C). Concentrations of C3G were generally higher in SCU212 than in SCU254 at all grain development stages beyond 5 DAF. At 35 DAF, the concentration of C3G in SCU212 rice grains (~ 500 mg/100 g DW) was five-fold higher than in SCU254 (~100 mg/100 g DW) (Fig 3A). Similarly, P3G concentration was also higher in SCU212 (around 50 mg/100 g DW) than in SCU254 (around 20 mg/ 100 g DW) at 35 DAF (Fig 3C).

The trend in rice grain C3G content was similar to that of C3G concentration over the course of grain filling in both genotypes, with a decline in grain C3G content from 20 to 30 DAF onwards in SCU212 and from 25 to 30 DAF in SCU254 (Fig 3A and 3B). Peak grain C3G content was ~20 mg/100 grains for SCU212 and ~12 mg/100 grains for SCU254. In contrast, grain P3G content peaked at ~1.6 mg/100 grains in both genotypes, and occurred at 15 and 20 DAF for SCU212 and SCU254, respectively. After 20 DAF, there was a rapid decline in P3G content in SCU254 grains to ~0.4 mg/100 grains at maturity, compared to ~0.9 mg/100 grains in SCU212 at maturity (Fig 3D).

Concentrations of γ-oryzanol in grains were higher in SCU212 than SCU254 at every grain development stage beyond 5 DAF (Fig 3E). At maturity, grains of SCU212 had a total γ-oryzanol concentration of approximately 65 mg/100 g dry weight (DW) compared to 50 mg/100 g DW in SCU254 (Fig 3E). Grain γ-oryzanol content peaked in both genotypes at 25 DAF, and at maturity both genotypes contained around 1.2 mg/100 grains (Fig 3F).

### Regulatory gene expression for anthocyanin biosynthesis

**Fig 4:**
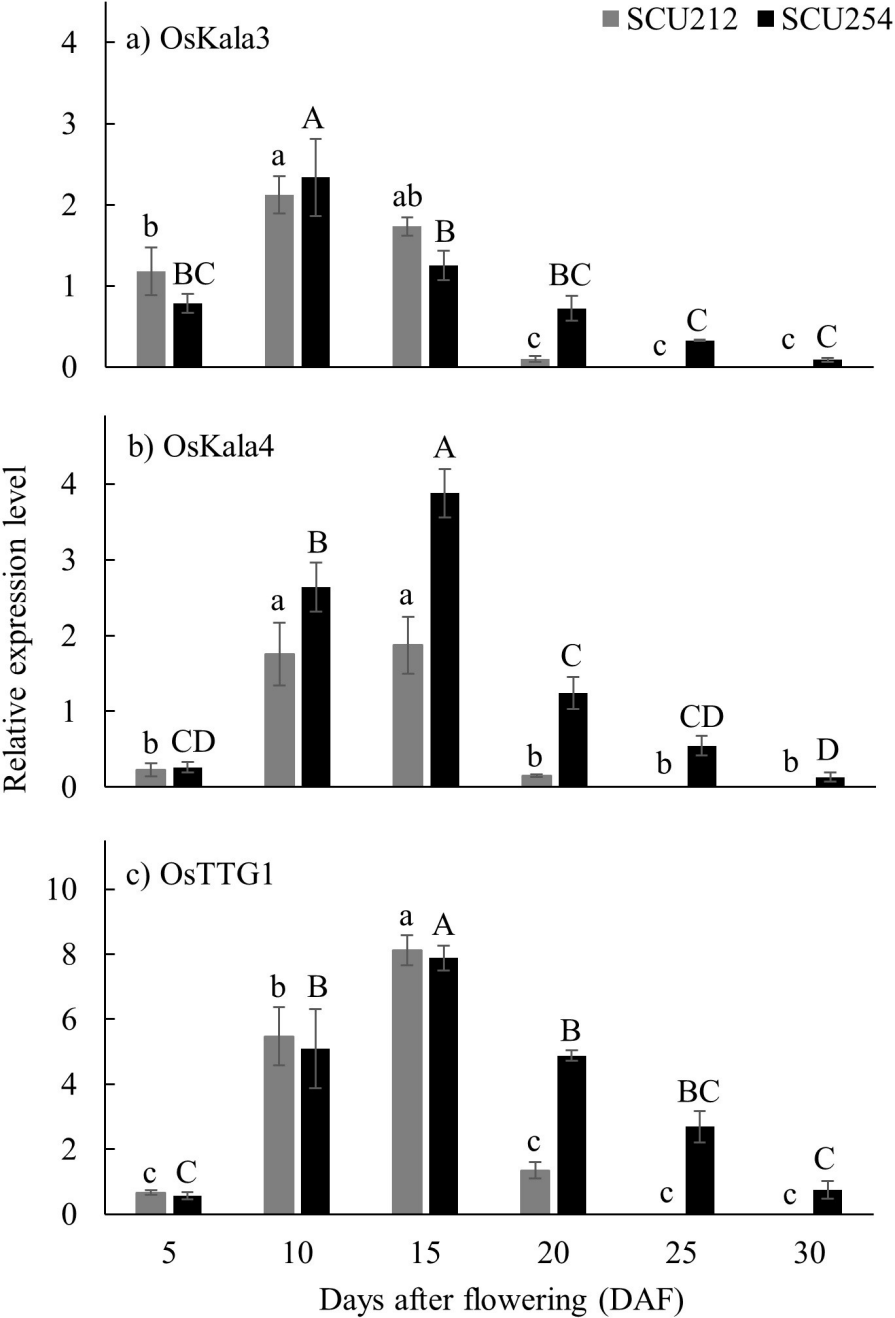
Relative expression level of genes involved in the MBW complex for anthocyanin regulation in rice genotypes using 2^-ΔΔ*CT*^ a) OsKala3, b) Oskala4, c) OsTTG1 expression levels. Results represent mean values ± SE from three independent biological replicates. Means for a given genotype that do not share a common letter are significantly different (*p*<0.05). The grey bar represents observations for genotype SCU212, and the black bar represents observations for SCU254.

Grain anthocyanin concentrations and the relative expression of three regulatory genes (OsKala3, OsKala4, and OsTTG1) were analysed over the time course of grain filling to investigate the relationship between the MBW complex genes and anthocyanin accumulation in two distinct black rice genotypes. OsKala4 is reported to be a positive regulator of anthocyanin accumulation and has a similar expression pattern to OsKala3 [10]. In our study, we found that the expression profiles of OsKala4 and OsTTG1 transcripts were similar during grain development, while OsKala3 displayed a different expression pattern (Fig 4). The relative expression of OsKala3 peaked before the peak expression level of OsKala4 and OsTTG1. In general, transcript levels of OsKala3 were highest at 10 DAF. In contrast, transcript levels of OsKala4 and OsTTG1 were highest at 15 DAF (Fig 4). OsKala3 expression was closely linked to anthocyanin content of SCU254 and SCU212 as its expression peaked just before the peak anthocyanin. The transcript level of OsTTG1 was highest compared to OsKala3 and OsKala4 throughout all the grain development stages except at 5 DAF, where the transcript level of OsKala3 was higher than OsTTG1.

Once the expression level of each regulatory gene in the MBW complex peaked (10 to 15 DAF), expression of the regulatory genes was downregulated as grains continued to develop further. When the relative expression level of all three regulatory genes was compared at 15 and 20 DAF for both genotypes, the decrease in the relative expression for all three regulatory genes between these two stages was more than 85% for SCU212. In contrast, in SCU254, the reduction in the relative expression of OsKala3 (~24%), OsKala4 (~64%), and OsTTG1 (~37%) was much lower.

## Discussion

Anthocyanin accumulation patterns and the corresponding regulation of those genes involved in the biosynthesis of this pigment in coloured rice during grain filling are poorly understood. Relevant literature is often contradictory, ranging from reports of a continual increase in anthocyanins during grain filling [4, 10, 23] to initial increases in concentration followed by a decrease as the grain nears maturity [11, 12]. In contrast, anthocyanins peaked at the fully ripe stage (30 DAF) in several rice genotypes assessed by Kim et al. (2021). While the differences observed between these studies might be due to the use of different genetic material and sampling technique, there is also a lack of adequate information on grain moisture and biomass accumulation. Without these data, it is difficult to determine whether changes in anthocyanin concentrations throughout grain development result from ‘dilution’ as grain weight increases or reflect metabolism of anthocyanins as the grain desiccates in the latter stages of grain filling.

In the present study, concentrations of two major anthocyanins of black rice grains, C3G and P3G, peaked at the dough grain stage. For SCU212 it was at 15 DAF while for SCU254 it was at 20 DAF. Anthocyanins then significantly declined as grains matured to around 500 mg/100 g DW in SCU212 and around 100 mg/100 g DW in SCU254. The C3G concentration in SCU212 was similar to the maximum total anthocyanin concentration in black rice reported in the literature of around 500 mg/100 g DW, usually around 30 DAF [12]. During the second half of grain filling, as the grain reached the fully mature stage, C3G concentrations decreased by more than 50% in both genotypes. Since dry grain weight continued to increase until 20 DAF (SCU212) or 25 DAF (SCU254), the initial decrease in anthocyanin concentrations from 15 DAF-20 DAF in SCU212 and from 20 DAF-25 DAF in SCU254 may be the result of the ‘yield dilution’ effect as suggested by Chen, Huang [11], where the decrease in anthocyanin concentration can be attributed to the increase in grain weight rather than an actual decline in grain anthocyanin. However, the decline in grain total anthocyanin content during the latter half of grain filling, when grain weight was constant (Fig 2), cannot be attributed to dilution and thus might indicate *in planta* decline in anthocyanins in rice grains, as reported for other horticultural crops [24]. Xie, Liu [25] reported pre-harvest anthocyanin degradation in grape skin at the late ripening stage, possibly caused by increased activity of peroxidase, β-glucosidase and polyphenol oxidase. The decline in anthocyanin content in grains at later stages of development may result from structural modification of anthocyanins, enzymatic and/or non-enzymatic degradation, combined with reduced production of anthocyanin through the downregulation of anthocyanin regulatory genes. Further study to investigate the reason for anthocyanin decline before grain maturity is warranted.

The regulatory genes of the MBW complex control the expression of structural genes for anthocyanin biosynthesis [10]. We found that expression of all three regulatory genes in the MBW complex, i.e., OsKala3, OsKala4, and OsTTG1, were upregulated during early grain development, corresponding to increases in anthocyanin content in the grain. OsKala3 expression in both genotypes peaked at 10 to 15 DAF before maximum anthocyanin content (15 to 20 DAF), while expression of OsKala4 and OsTTG1 peaked later at 15 to 20 DAF at maximum anthocyanin content. This result differed from Kim, Yang [10], who reported that all three regulatory genes peaked at the same time point (20 DAF) before the peak in anthocyanin content (35 DAF) in the black rice genotypes used in their study. The finding that OsKala3 expression precedes OsKala4 and OsTTG1 expression in the rice grain might indicate that OsKala3 regulates the expression of OsKala4 and OsTTG1. The bHLH TF, OsKala4, was thought to be the only pericarp-specific anthocyanin regulator in rice [3]. Recently Kim, Yang [10] demonstrated the importance of the MBW TF (OsKala3) along with OsKala4 and OsTTG1 for anthocyanin biosynthesis in the rice pericarp. There have been numerous reports across different crops and tissues that suggest a possible transcriptional hierarchy between regulatory genes of the MBW complex. While in rice culms it was suggested that bHLH could activate the expression of the MYB gene [17], bHLH expression was controlled by MYB TF in Arabidopsis roots [26]. Moreover, Sun, Zhang [17] also identified a bHLH binding site in the MYB promoter and a MYB binding site in the promoter of WD40 in rice culms, suggesting that bHLH plays a crucial role in the regulatory cascade of the MBW complex. In petunia leaves, bHLH expression was induced by ectopic expression of MYB, while in anthers, the role was suspected to be reversed, with MYB expression controlled by bHLH [27]. Consequently, the transcriptional hierarchy of MBW complex expression might differ between plant species and tissues. Further studies are needed to explore the potential transcriptional hierarchy among regulatory genes in the MBW complex of the pigmented rice pericarp, and in particular, a wider range of rice genotypes should be examined.

When rice grains attained maximum anthocyanin levels (around 15–20 DAF), MBW was significantly downregulated in both rice genotypes. Kim, Yang [10] reported a statistically significant but slight decrease in transcripts for regulatory and structural genes at peak anthocyanin levels. Still, the study did not continue to monitor gene expression patterns over time and, equally important, lacked rice grain physiological data to confirm that the grain was harvested at full maturity. Our study suggests that anthocyanin production is not sustained throughout grain development in black rice, and anthocyanin biosynthesis stops before full grain maturity is reached. C3G content decreased more rapidly in SCU254 as compared to SCU212 (63% decrease in C3G content in SCU212 vs. 82% decrease in SCU254) (Fig 3B). In contrast, MBW complex genes were downregulated more quickly in SCU212 than in SCU254 (Fig 4). However, SCU254 grains dried at a slower rate relative to those of SCU212 (Fig 1C). Since water activity (i.e., water available for reaction) determines the enzymatic and non-enzymatic browning of the grain as a result of anthocyanin degradation [28], the difference in drying rate and moisture content of the two genotypes might explain higher anthocyanin decline in SCU254, irrespective of more sustained MBW gene expression. These data suggest that these two particular genotypes might vary in anthocyanins decline patterns. Further studies are warranted to investigate the possible cause of the decrease in anthocyanins and characterise any genetic variation in the anthocyanin decrease in rice grains. A comparison of these two major anthocyanins revealed that the decline in C3G (non-methylated anthocyanin) concentration (~63% in SCU212 and ~82% in SCU254) was more pronounced than that of P3G (methylated anthocyanin) concentration (~50% in SCU212 and 75% in SCU254) in both genotypes, suggesting that P3G was more stable than C3G. A similar finding was reported by Xie, Liu [25] where non-methylated anthocyanin derivates degraded at a higher rate than methylated anthocyanins in a red flesh grape variety at a late stage of fruit development. Because of the relative stability of P3G as compared to C3G, it might be worth screening a larger pool of pigmented rice genotypes for those that contain a relatively high concentration of P3G.

Total γ-oryzanol in the genotypes SCU212 and SCU254 ranged from 50 to 60 mg/100 g DW of grain, similar to reports for other rice varieties [21]. While studies by Chen and Bergman [15] and Lin and Lai [13] reported an increase in γ-oryzanol with grain maturity in different white rice genotypes, Kim, Kim [14] reported a decrease in γ-oryzanol towards maturity in ‘Chuchung’. However, none of these studies examined γ-oryzanol concentrations during grain development from the early stage to full maturity, and they lack physiological data at the different grain development stages. In this study, total rice grain γ-oryzanol concentration and content increased until 25 DAF in SCU212 and 30 DAF in SCU254 and remained stable as the grain matured (Fig 3E and 3F). Anthocyanins are water-soluble compounds stored in cell vacuoles with several peroxidase enzymes known to oxidise phenolic compounds [7]. Anthocyanins have poor stability and are susceptible to various non-enzymatic factors such as pH, light and temperature [29]. In contrast, γ-oryzanol are mostly lipophilic in nature and found to be more thermostable than anthocyanins [7, 30]. While their subcellular localisation remains unclear, *in planta* decline of γ-oryzanol has not been previously reported.

## Conclusion

In this study, anthocyanins tended to peak at the dough stage and decrease before reaching the fully ripe stage, whereas γ-oryzanol increased continually throughout grain development. Although anthocyanins were highest at the dough stage, harvesting at this stage is not ideal because of high grain moisture content which will affect quality during long-term storage. Hence, it is pertinent to identify the underlying causes for the decline in anthocyanin content so harvest can be carried out at a later stage of maturity without compromising anthocyanin or γ-oryzanol content. Our results also reveal that the decline in anthocyanin content is genotype specific, and the decline in C3G content is more pronounced than P3G. This study further confirmed that three key regulatory genes (OsKala3, OsKala4, and OsTTG1) in the MBW complex for anthocyanin biosynthesis showed down-regulation after the dough stage, corresponding with a decrease in anthocyanin content prior to maturity. However, this finding needs to be validated across a much wider range of black rice genotypes.

## Supporting information

S1 Fig De-husked rice grains. Harvested from tagged panicles of the genotypes SCU 212 and SCU254 at 5, 10, 15, 20, 25, 30 and 35 Days after flowering (DAF).
(DOCX)

S1 Table Black rice genotypes (*Oryza sativa*) used for the preliminary experiment. (DOCX)

S2 Table Key chemical properties of the soil used in the gain development experiment. All values are means of duplicate samples measured by the Environment Analysis Laboratory at Southern Cross University, Lismore.
(DOCX)

S3 Table Primers sequence for regulatory genes. Forward (F) and Reverse (R) primer sequences of three regulator genes of the MBW complex and internal reference used for qPCR.
(DOCX)
