# Supplementary material for: Accumulation patterns of anthocyanin and γ-oryzanol during black rice grain development
Source: PLoS One. 2024 May 22;19(5):e0302745. doi: 10.1371/journal.pone.0302745 (PMC11111080; doi:10.1371/journal.pone.0302745)
Supplement: S1 Fig — Harvested from tagged panicles of the genotypes SCU 212 and SCU254 at 5, 10, 15, 20, 25, 30 and 35 Days after flowering (DAF). (DOCX) [file pone.0302745.s001.docx]

**
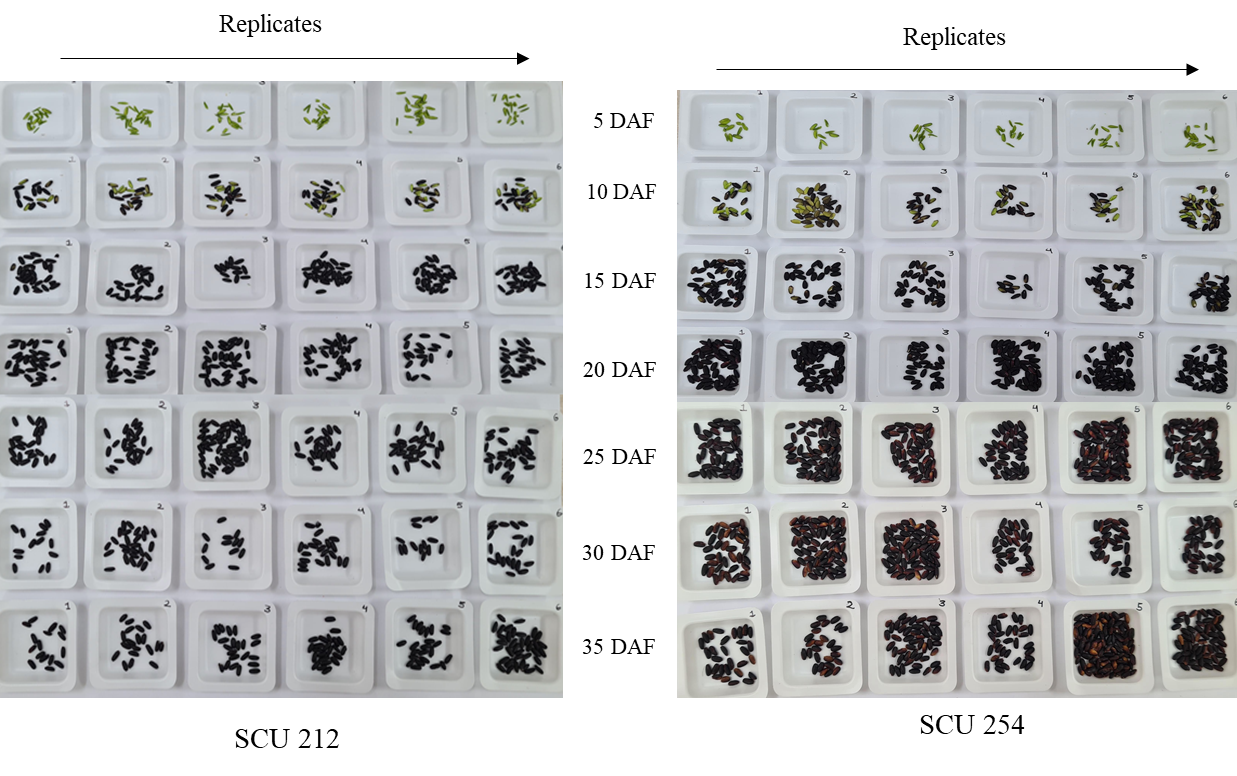
**

**S1 Fig. Dehusked rice grains.** Harvested from tagged panicles of the genotypes SCU 212 and SCU254 at 5, 10, 15, 20, 25, 30 and 35 Days after flowering (DAF)


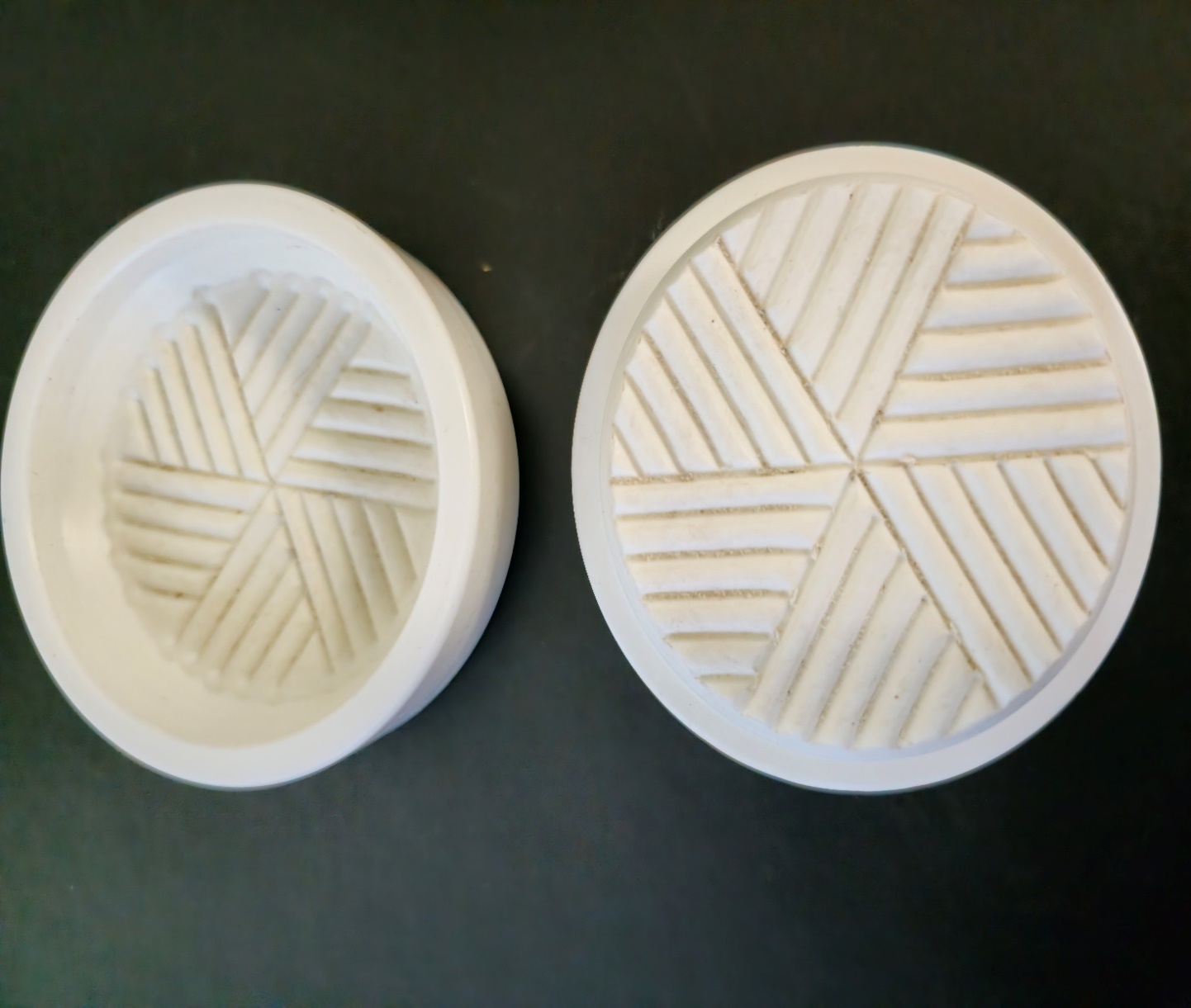


**S2 Figure:** Manual dehusker used for dehusking black rice
