# Supplementary material for: Accumulation patterns of anthocyanin and γ-oryzanol during black rice grain development
Source: PLoS One. 2024 May 22;19(5):e0302745. doi: 10.1371/journal.pone.0302745 (PMC11111080; doi:10.1371/journal.pone.0302745)
Supplement: S1 Table — (DOCX) [file pone.0302745.s002.docx]

**S1 Table.** **Black rice genotypes (*Oryza sativa*) used for the preliminary experiment.**

| SCU | Plant_line_name | Plant Height (cm) | Description | Country | Photo |
| --- | --- | --- | --- | --- | --- |
| 19 | CHENLUN (HEA LI) 55 | 125 | Medium red black | China | 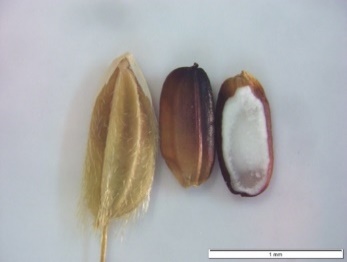 |
| 26 | PULUT LAPAUNG | 115 | Medium black | Indonesia | 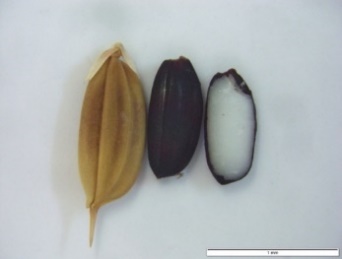 |
| 29 | ADONG | 118 | Medium red black | Malaysia | 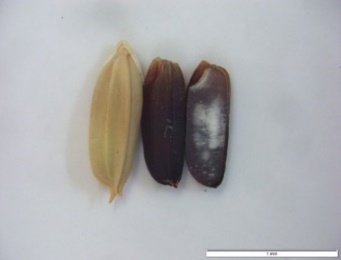 |
| 63 | KEAREN HITAM | 124 | Medium slender black | Indonesia | 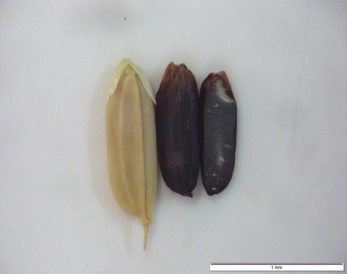 |
| 136 | MEI SHAN HEI KU | 115 | Short bold black | china | 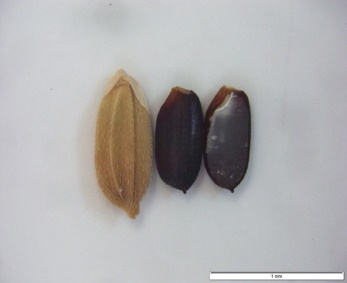 |
| 137 | HEI MI CHAN | 115 | Medium bold black | China | 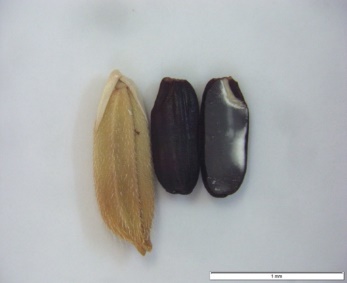 |
| 138 | WC 603 | 110 | Medium black | China | 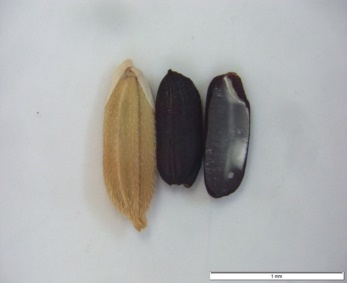 |
| 178 | PUTAN SUNDIG | 115 | Medium black | Malaysia | 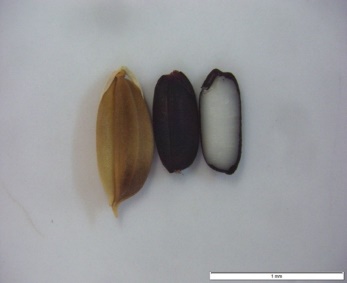 |
| 211 | DPRK 036S 7220 | 97 | Short bold black | North Korea | 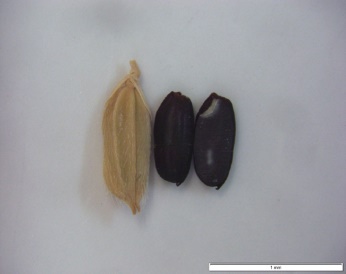 |
| 212 | DPRK 036S 9917 | 110 | Short black | North Korea | 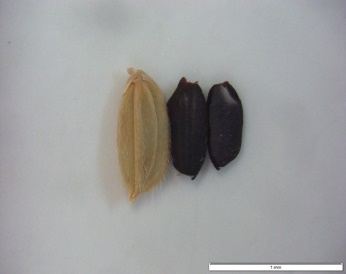 |
| 244 | DPRK 036S 474 | 97 | Short bold black | North Korea | 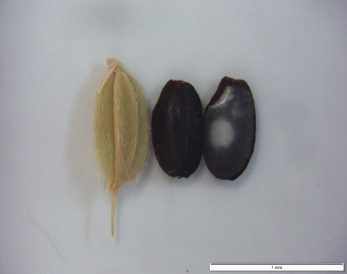 |
| 111 | PUTAN ITUM | 107 | Medium black | Philippines | 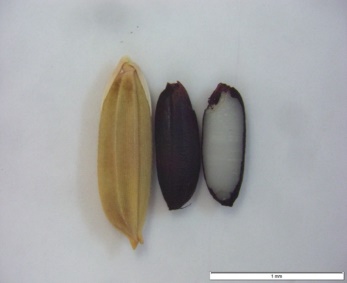 |
| 256 | 861 30-45 B (COLORED) | 65 | Short bold black | China | 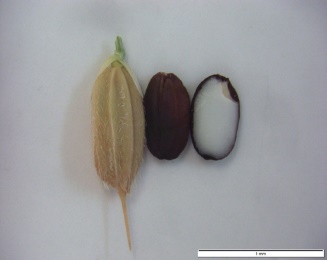 |
| 254 | 861 30-45 C (COLORED) | 65 | short bold black | China | 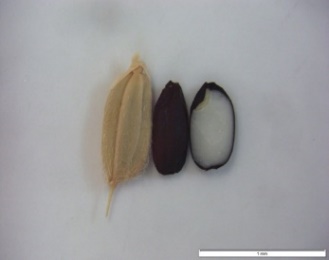 |
