# Supplementary material for: Accumulation patterns of anthocyanin and γ-oryzanol during black rice grain development
Source: PLoS One. 2024 May 22;19(5):e0302745. doi: 10.1371/journal.pone.0302745 (PMC11111080; doi:10.1371/journal.pone.0302745)
Supplement: S2 Table — All values are means of duplicate samples measured by the Environment Analysis Laboratory at Southern Cross University, Lismore. (DOCX) [file pone.0302745.s003.docx]

**S2 Table.** **Key chemical properties of the soil used in the gain development experiment.** All values are means of duplicate samples measured by the Environment Analysis Laboratory at Southern Cross University, Lismore.

| **Soil Property** | **Mean** |
| --- | --- |
| Total Carbon (%) | 4.21 |
| Total Nitrogen (%) | 0.29 |
| Bray 1 Phosphorus (mg/kg) | 6.24 |
| pH (1:5 water) | 5.10 |
| Electrical Conductivity (dS/m) | 0.072 |
| Effective Cation Exchange Capacity (ECEC) (cmol+/kg) | 15.49 |
| *Base Saturation* |  |
| Calcium (%) | 52.55 |
| Magnesium (%) | 36.86 |
| Potassium (%) | 2.87 |
| Sodium - ESP (%) | 1.09 |
| Aluminium (%) | 2.02 |
| Hydrogen (%) | 4.56 |
| *DTPA extractable micronutrients* |  |
| Zinc (mg/kg) | 1.34 |
| Manganese (mg/kg) | 21.57 |
| Iron (mg/kg) | 215.61 |
| Copper (mg/kg) | 0.38 |
