# Supplementary material for: Accumulation patterns of anthocyanin and γ-oryzanol during black rice grain development
Source: PLoS One. 2024 May 22;19(5):e0302745. doi: 10.1371/journal.pone.0302745 (PMC11111080; doi:10.1371/journal.pone.0302745)
Supplement: S3 Table — Forward (F) and Reverse (R) primer sequences of three regulator genes of the MBW complex and internal reference used for qPCR. (DOCX) [file pone.0302745.s004.docx]

**S3 Table. Primers sequence for regulatory genes.** Forward (F) and Reverse (R) primer sequences of three regulator genes of the MBW complex and internal reference used for qPCR

| Regulatory genes | OsKala3-F | CACCACCAGTAGGAGGAGGA |
| --- | --- | --- |
|  | OsKala3-R | TTCGATTCCACAATGATCCA |
|  | OsKala4-F | CCGAGAGAAGCTCAACGAGA |
|  | OsKala4-R | TGCAAGTATGGATGCCTTGT |
|  | OsTTG1-F | CAGCTTACCCGGAGTGGATA |
|  | OsTTG1-R | CCAAGGCTTCAGAGTCGAAC |
| Housekeeping gene | OsUBI-F | GAAGTAAGGAAGGAGGAGGA |
|  | OsUBI-R | AAGGTGTTCAGTTCCAAGG |
